# Partial Nephrectomy of Renal Cell Carcinoma in Horseshoe Kidney with Assistance of Intraoperative Retrograde Pyelogram: A Case Report and Literature Review

**DOI:** 10.15586/jkc.v13i1.449

**Published:** 2026-03-23

**Authors:** Omar Wai-kiu Tsui, Sunny Shek-long Tsang, Henry Hong-yin Lie, Thomas Ting-fung Wong, Wilson Pui-long Hung, Simon Hoi-lung Wong, Terence Chun-ting Lai, Chiu-fung Tsang, Ada Tsui-lin Ng

**Affiliations:** Division of Urology, Department of Surgery, Queen Mary Hospital, the University of Hong Kong, Hong Kong

**Keywords:** horseshoe kidney, partial nephrectomy, renal cell carcinoma, retrograde pyelogram, three-dimensional reconstruction

## Abstract

Horseshoe kidney (HSK), characterized by the fusion of two kidneys forming a U-shape, presents intricate challenges in renal anatomy and poses a unique landscape for the development of renal cell carcinoma (RCC). This abstract delves into the case of a 74-year-old male with HSK who also developed RCC, where the employment of intraoperative retrograde pyelogram (RP) played a pivotal role in enhancing surgical precision. The patient’s complex tumor was successfully resected through meticulous identification and dissection. A comprehensive literature review reveals the significance of laparoscopic and robotic surgeries in treating RCC within HSKs, with 3D-reconstruction aiding in surgical planning. While advancements in imaging technologies have improved surgical outcomes, the underexplored utility of intraoperative RP stands out. RP provided real-time insights into the renal pelvis anatomy, guiding the surgical team in navigating intricate structures and ensuring optimal reconstruction post-tumor excision. The discussion underscores the challenges posed by RCC in HSKs, importance of preoperative 3D-reconstruction and angiography in surgical planning, and the critical role of intraoperative RP in mapping renal pelvis anatomy. Unlike conventional imaging methods, RP offers dynamic visualization of the renal drainage system, safeguarding against inadvertent closures and enhancing surgical precision. The successful utilization of RP in this case not only facilitated safe tumor resection but also highlighted its potential in managing unclear renal structures. In conclusion, the integration of intraoperative RP in surgical interventions for RCC within HSKs proves instrumental in enhancing surgical precision and navigating complex anatomical variations. By emphasizing the importance of real-time imaging guidance, surgeons can optimize treatment outcomes for individuals with RCC in the challenging context of HSK anomalies.

## Introduction

Horseshoe kidney (HSK), a congenital anomaly where the two kidneys are fused at the lower ends forming a U-shape, presents a unique challenge in renal anatomy. This condition, arising during fetal development, can result in a range of potential complications affecting kidney function and overall urological health. With an estimated prevalence of about 0.25% among renal anomalies, HSK stands as a relatively rare occurrence (1). Research suggests a higher incidence in males compared to females, with a male-to-female ratio of approximately 2:1 (2). Though many individuals with this condition remain asymptomatic throughout their lives, some may encounter complications such as kidney stones, urinary tract infections, or structural abnormalities that require medical attention (3). Given the complexity and potential ramifications of HSK, close monitoring and management are crucial. Protection of remaining renal function in the solitary kidney is of utmost importance functionally in patients, while the oncological outcome of tumor resection is also crucial. Hence, image-guided preoperative assessments and intraoperative retrograde pyelogram (RP) plays important roles in achieving these aims.

## Case Report

**Figure 1:**
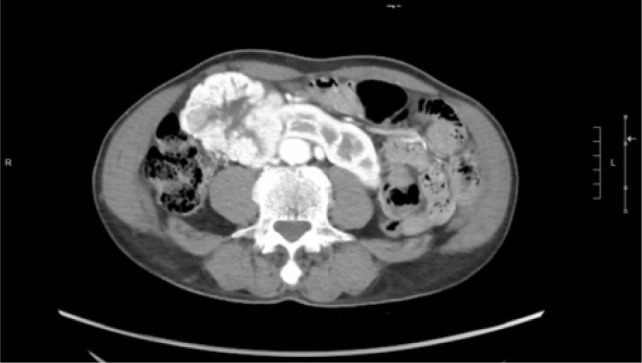
Axial cut of arterial phase-enhanced CT abdomen at the tumor level.

**Figure 2:**
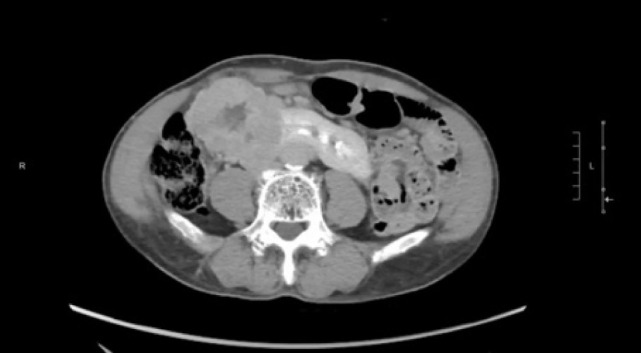
Axial cut of delayed phase-enhanced CT abdomen at the tumor level.

**Figure 3:**
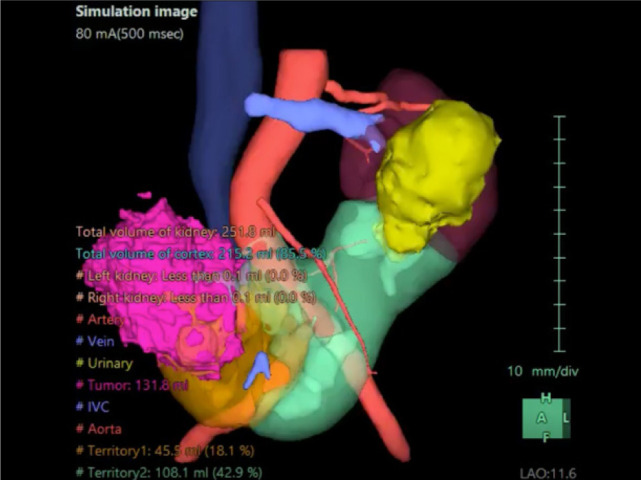
3D reconstruction of horseshoe kidney and the tumor.

A 74-year-old Chinese male patient, ex-smoker, and social drinker, with a history of carcinoma of prostate in remission with laparoscopic radical prostatectomy done 11 years ago presented with an incidental finding of a right abdominal mass without frank symptoms. Preoperative eGFR ranges from 80 to 90 mL/min/1.73m^2^. Computed tomography (CT) showed a 7 cm heterogeneous mass on the right side of the HSK with complex hilar anatomy (Figures 1 & 2). Dual tracer (C-11 acetate and F18 FDG) positron emission tomography (PET-CT) showed moderate and mildly avid renal cell carcinoma (RCC) arising from HSK, without nodal or distant metastases. A 3D image reconstruction using FUJIFILM Synapse system was performed, showing complex tumor and vascular anatomy (two renal arteries and an additional artery to isthmus) (Figure 3). An open partial right nephrectomy was planned.

**Figure 4:**
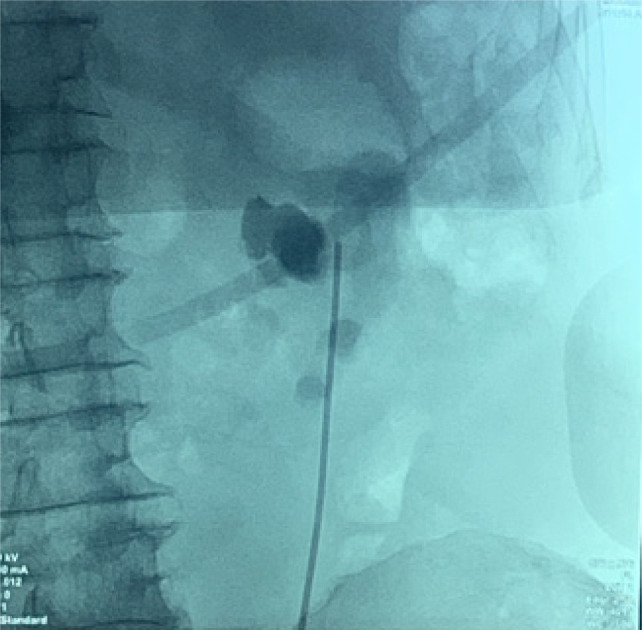
Left renal pelvis.

**Figure 5:**
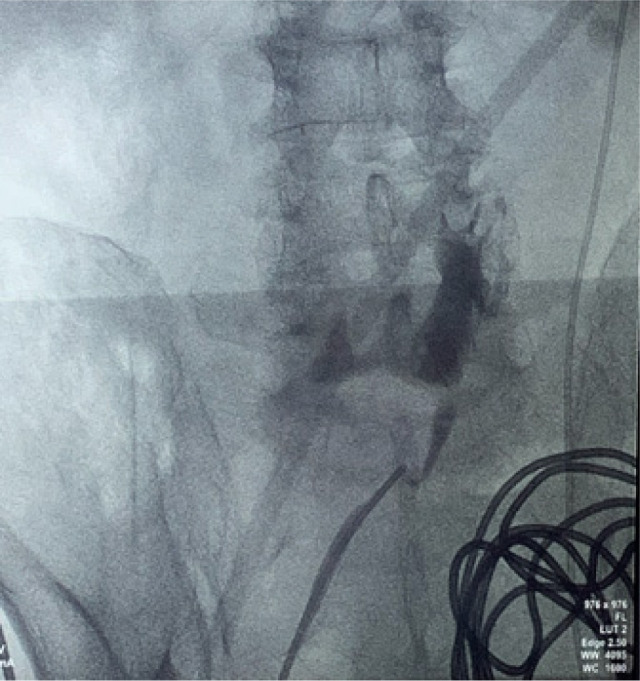
Right renal pelvis.

**Figure 6:**
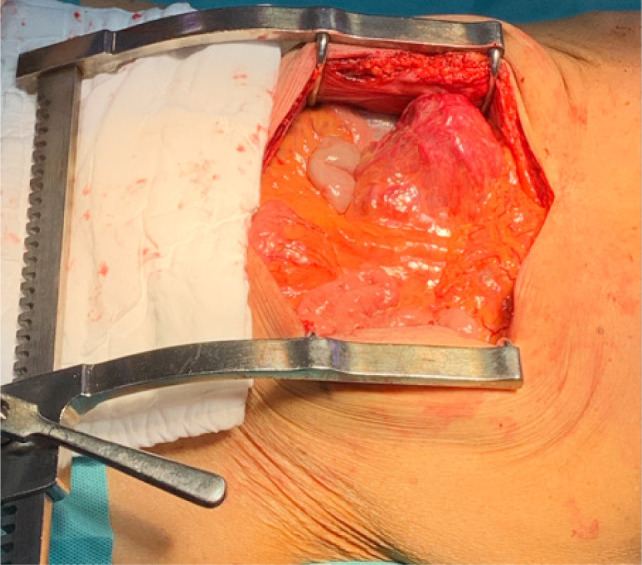
Tumor before resection.

**Figure 7:**
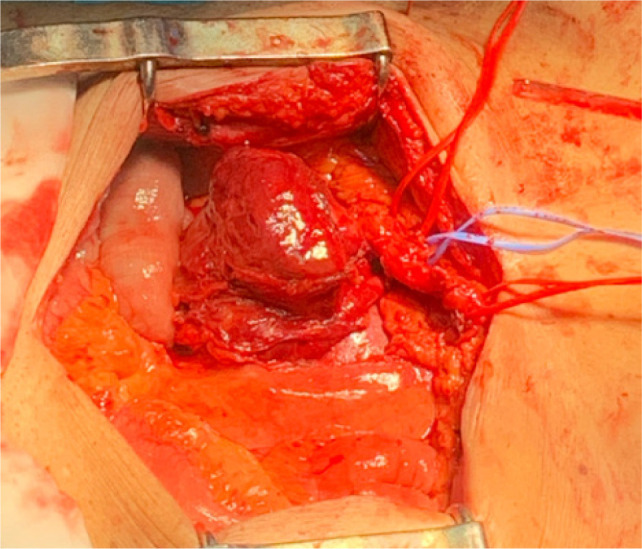
Carefully slung vasculatures.

**Figure 8:**
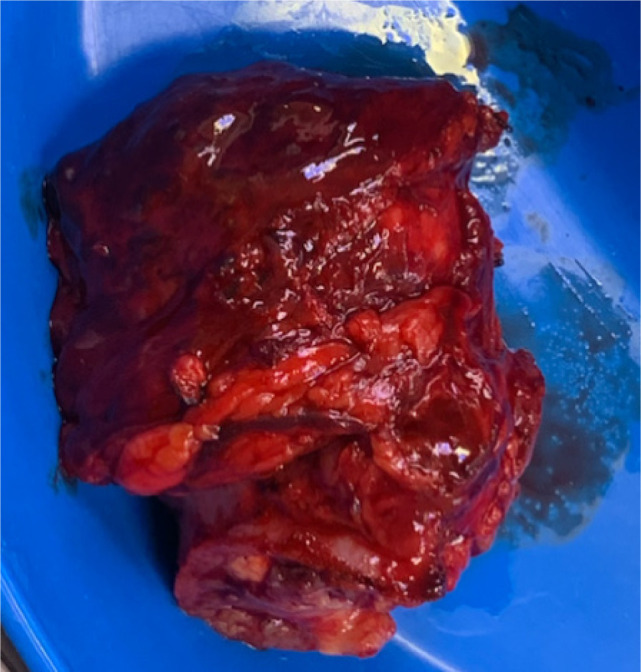
Resected tumor.

Bilateral RP and ureteric catheterization were first performed for the identification of the hila and calyces of the HSK (Figures 4 & 5). A lower midline laparotomy was performed. The tumor was identified, measuring 7 cm in diameter (Figure 6). Careful dissection of complex hilar structure was performed. Two renal arteries and two renal veins supplying the right side were identified and slung (Figure 7). The tumor was resected with artery clamping (Figure 8). The right lateral calyx of the pelvicalyceal (PC) system was involved and resected. The PC system was closed with 3/O PDS. Renorrhaphy was completed with 3/O V-Loc, and parenchyma was closed with 2/O V-Loc. Ischemic time was 20 minutes, and total operation time was 332 minutes. Blood loss was 420 mL. The patient had completed 1 week of levofloxacin and was discharged on postoperative day 6 with one kick of fever that spontaneously subsided.

Pathology report shows a 7.5 × 7 × 6.5 cm tumor of 113.2 g. On macroscopic examination, there were areas of hemorrhage and cystic change without gross perinephric tissue involvement. Microscopic examination shows clear cell RCC of WHO grade 2, with a pathological T2a staging. Surgical margin is clear.

Patient was followed up in 4 months’ time, and renal function was normal with no recurrence detected.

## Literature review

We have reviewed 47 patients with renal tumor in HSK from multiple case reports; laparoscopic and robotic surgeries have similar clinical outcomes to open partial nephrectomy. Twenty patients (42.6%) are female, and 27 patients (57.4%) are male. The mean age is 53.93 years old (standard deviation (SD) 19.20 years old). The mean operative time is 197.85 minutes (SD 51.90 minutes), the mean ischemic time is 23.60 minutes (SD 10.77 minutes), and the mean blood loss is 235.56 mL (SD 172.35 mL). The average length of stay is 4.4 days (SD 2.70 days). Twenty patients (46.8%) have preoperative 3D reconstruction. Twenty-nine patients have RCC, five patients have primary carcinoid tumor, two patients each have oncocytoma and Wilm’s tumor, and one patient each has squamous cell carcinoma, angiomyolipoma, and hemangioma. In eight patients, the tumor type is not mentioned. Details of each case are presented in Table 1.

**Table 1:** Literature review on 3D preoperative reconstruction and intraoperative retrograde pyelogram.

PMID	Age	Sex	Robot/Lap/Open/Nil	3D recon.	Intraop RP	Operative time (mins)	Ischemic time (mins)	Blood loss (mL)	POD discharge	Complication	Tumor type
15679967	26	F	Open	N	N		30			No recurrence at 8 months	Clear cell RCC
16230165	52	M	Open	N	N	165		400			
17110020	62	F	Laparoscopy	N	N	210		70	3		Oncocytoma
17874547	52	F	Open	N	N						Angiomyolipoma
19523243	50	F	Nil	N	N						Primary carcinoid tumor
19588864	31	M	Nil	N	N						Primary carcinoid tumor
19926945	59	M	Nil	Y	N						Clear cell RCC
20801492	69	M	Nil	N	N						SCC
22195272	21	F	Laparoscopy	Y	N	186	28	490			
26766809	42	F	Nil	Y	N					No recurrence at 7 months	Clear cell RCC
26892057	53	M	Open, subcostal	N	N				2		Clear cell RCC
26892057	49	F	Open, subcostal	N	N				2		Papillary RCC
26892057	50	F	Open, subcostal	N	N				2		Primary carcinoid tumor, adenoCA
26892057	56	M	Laparoscopy	N	N				4		Clear cell RCC
26892057	59	F	Open, midline	N	N				7		Clear cell RCC
26892057	86	F	Open, midline	N	N				5		Clear cell RCC
26892057	60	M	Open, midline	N	N				7		Clear cell RCC
26892057	66	M	Open, midline	N	N				3		Clear cell RCC
26948529	5	F	Open	N	N						Wilm’s tumor
28245540	79	M	Open	Y	N	200	12	380			Clear cell RCC
28435792	69	M	Laparoscopy	Y	N	156	24	75	4	No recurrence at 6 months	Clear cell RCC
29288785	53	F	Robot-assisted laparoscopy	Y	N	170		150	2		Oncocytoma
29473385	37	F	Open	N	N						Clear cell RCC
29977636	83	F	Laparoscopy	N	N	159	11		11	No recurrence at 43 months	Clear cell RCC
30357753	51	F	Laparoscopy	N	N						Primary carcinoid tumor
31808400	53	M	Laparoscopy	Y	N						RCC
32489122	65	M	Laparoscopic	Y	N	210	30	370		No recurrence at 24 months	Clear cell RCC
32743445	66	M	Robot-assisted laparoscopy	Y	N	295	13	100	7	No recurrence at 6 months	Clear cell RCC
32775638	55	F	Laparoscopy	Y	N	208		200		No recurrence at 3 months	Clear cell RCC
32775638	50	M	Laparoscopy	Y	N	225	28	350		No recurrence at 12 months	Clear cell RCC
33990296	50	M	Open	Y	N (used USG)					No recurrence at 6 and 12 months	RCC (grade II)
34258538	84	F	Robot-assisted laparoscopy	Y	N	185	18		8		Clear cell RCC
34326031	82	M	Laparoscopy	Y	N				2		
34326031	64	M	Laparoscopy	Y	N				2		
34521468	9	M	Nil	N	N						Wilm’s tumor
35725279	60	M	Laparoscopy	N	N		53				Chromophobe RCC
35727421	71	F	Laparoscopy	Y	N	135	25	80	6		Hemangioma
35795128	75	M	Laparoscopy	N	N	225	18			No recurrence at 3 months	Clear cell RCC
37275562	19	M	Robot-assisted laparoscopy	N	N	140		170	3		SDH-deficient RCC
37275562	19	M	Robot-assisted laparoscopy	N	N	160		600	1		SDH-deficient RCC
37908786	41	M	Open	N	N (used USG)	215		200	7	No recurrence at 16 months	Clear cell RCC
38178790	74	M	Robot-assisted laparoscopy	Y	N						
38233275	42	M	Laparoscopy	Y							RCC
38711819	65	F	Robot-assisted laparoscopy	Y	N	355	19			No recurrence at 6 months	Primary carcinoid tumor
38936940	61	M	Robot-assisted laparoscopy	Y	N	181	14	100		No recurrence at 53 months	Clear cell RCC
38936940	45	F	Robot-assisted laparoscopy	Y	N	177	31	34		No recurrence at 13 months	Clear cell RCC
39914014	65	M	Nil	Y	N					No recurrence at 52 months	

Note: Y = yes; N = no; Lap = laparoscopy; Nil = not mentioned; Intraop RP = Intraoperative retrograde pyelogram; 3D recon. = 3-dimensional reconstruction; POD discharge = postoperative day discharge; RCC = renal cell carcinoma; SCC = squamous cell carcinoma.

Based on the findings from the literature review, laparoscopic and robotic surgeries have emerged as sophisticated approaches for treating tumors in HSKs. Notably, approximately half of the cases incorporate 3D-reconstruction, underscoring the established efficacy of this technique in orchestrating surgeries involving intricate anatomy. However, despite these advancements, none of the studies have documented the utilization of intraoperative retrograde pyelography (RP). In contrast, RP offers real-time insights into the renal pelvis anatomy and its drainage system, equipping surgeons with invaluable details on the complexities of renal pelvis anatomy. This dynamic tool not only furnishes immediate feedback on the viability of renal pelvis reconstruction post-tumor excision but also averts the risk of inadvertent closure of the drainage system following reconstruction, thereby enhancing surgical precision and postoperative outcomes.

## Discussion

RCC is the most common (around 90%) type of kidney cancer and can arise within the renal tissue of an HSK (4). While upper tract transitional cell carcinoma accounts for 5–10% of all transitional cell carcinoma, and renal sarcoma accounts for <1% of renal tumor (6). The challenges posed by RCC in individuals with HSK are similar to those faced by individuals with RCC in normal kidney anatomy. Early detection and appropriate management of RCC in individuals with HSK are essential to mitigate the risks associated with this complication and improve patient outcomes.

Surgical challenges encountered when treating RCC in a HSK can be notably complex, particularly due to the presence of aberrant vascular structures surrounding the tumor (7). To address these intricacies effectively, preoperative 3D reconstruction of the HSK is imperative to safeguard delicate structures during the surgical intervention (8), as well as preoperative angiography (9). Intraoperatively, the meticulous identification and careful dissection along these vascular structures with the usage of RP is paramount to prevent complications such as solitary kidney ischemia and potential risks like ischemic bowel.

HSK has variable arterial blood supply and vessel size. Injury to arteries and their branches in HSK can cause ischemia and deterioration in renal function (9). Pre-operative angiography is widely used among renal transplant cases and trauma patients, allowing urologists to visualize vessels to protect (10). Preoperative HSK angiography can aid surgical precision in terms of protection of significant vessels from being damaged. Other imaging modalities can also serve similar purposes, such as CT angiography (11). Along with 3D reconstruction, preoperative angiography can aid surgeons to remove the tumor precisely while not disturbing significant vessels, in order to achieve the highest remaining renal parenchyma (RRP), aiming more than 50% of the remaining HSK parenchyma (12).

Moreover, challenges may arise when conventional imaging methods such as CT scans and 3D reconstructions fail to provide a clear depiction of the renal pelvis, leading to uncertainties regarding its boundaries and internal structure. In such instances, intraoperative strategies become crucial, including RP of the renal pelvis and collecting system to guide surgical decision-making. In addition, the intraoperative injection of the dye can be utilized to confirm the precise location of the renal pelvis, aiding in surgical navigation. Real-time imaging techniques using fluoroscopy at key junctures in the procedure, such as before making incisions, prior to tumor removal, and after closing the renal pelvis, offer valuable insights and guidance in managing the unclear structures effectively. Employing on-table surgical planning based on real-time imaging data becomes essential in adapting to unforeseen challenges and ensuring optimal treatment outcomes for RCC within the unique anatomical context of an HSK.

We have effectively utilized intraoperative RP to enhance surgical precision in mapping the intricate anatomy of the renal pelvis. Unlike previous methods relying on CT scans and 3D reconstructions, which often fall short in detailing the exact drainage pathways from the renal pelvis to the ureters in cases of HSKs, intraoperative RP provides invaluable insights. This technique enables our surgical team to gain a comprehensive understanding of the renal drainage system, crucial for ensuring that the renal pelvis remains open and connected to the appropriate structures following partial nephrectomy. In essence, intraoperative RP serves as a vital and secure tool for urologists, empowering them to navigate the complexities surrounding anomalous renal pelvis structures with precision, thereby mitigating the risks of postoperative complications.

Preoperative 3D reconstruction and angiogram, along with intraoperative RP, provide the safest surgical planning and operation to patients, securing renal function, RRP, and ensuring oncological outcome.

## Conclusion

In conclusion, the intricate case of the 74-year-old male patient with HSK and RCC highlights the challenges and complexities faced in managing RCC within this congenital anomaly. Surgical interventions necessitated precise planning, meticulous intraoperative techniques, and utilization of advanced imaging modalities to navigate the unique anatomical intricacies of the HSK. The successful enucleation of the tumor amidst complex vascular structures and ambiguous renal pelvis anatomy underscores the importance of tailored approaches in addressing RCC within such atypical renal configurations. Intraoperative RP provides real-time image guidance for surgical safety and information of dynamic renal pelvis drainage system. Multidisciplinary collaboration, personalized care strategies, and real-time imaging guidance played pivotal roles in ensuring optimal surgical outcomes and effective management of RCC in this specialized context. By emphasizing a patient-centered approach and leveraging cutting-edge technologies, healthcare providers can enhance treatment efficacy and quality of care for individuals with RCC in the challenging setting of HSK anomalies.

## Mandatory Disclosure on Use of Artificial Intelligence

The authors declare that no AI-assisted tools were used in the preparation of this manuscript. All references have been manually verified for accuracy and relevance.
